# Subcritical Water Extraction of *Onosma mutabilis*: Process Optimization and Chemical Profile of the Extracts

**DOI:** 10.3390/molecules28052314

**Published:** 2023-03-02

**Authors:** Selda Doğan Çalhan, Bahar Meryemoğlu, Pelin Eroğlu, Barış Saçlı, Dimitrios Kalderis

**Affiliations:** 1 Department of Pharmaceutical Biotechnology, Faculty of Pharmacy, Mersin University, Mersin 33169, Turkey; 2 Central Research Laboratory, Cukurova University, Adana 01330, Turkey; 3 Department of Chemistry, Science Faculty, Mersin University, Mersin 33110, Turkey; 4 Department of Electronic Engineering, Hellenic Mediterranean University, 73100 Chania, Greece

**Keywords:** *O. mutabilis*, subcritical water, total phenol content, response surface methodology, green chemistry

## Abstract

The aboveground and root parts of *Onosma mutabilis* were extracted using subcritical water and the process was optimized with response surface methodology. The composition of the extracts was determined by chromatographic methods and compared to that of conventional maceration of the plant. The optimum total phenolic contents for the aboveground part and the roots were 193.9 and 174.4 μg/g, respectively. These results were achieved at a subcritical water temperature of 150 °C, an extraction time of 180 min, and a water/plant ratio of 0.1, for both parts of the plant. Principal component analysis revealed that the roots contained mainly phenols, ketones, and diols, with the aboveground part mostly alkenes and pyrazines, whereas the extract from maceration contained mainly terpenes, esters, furans, and organic acids. The quantification of selected phenolic substances showed that subcritical water extraction compared favorably to maceration, especially with respect to pyrocatechol (1062 as compared to 10.2 μg/g) and epicatechin (1109 as compared to 23.4 μg/g). Furthermore, the roots of the plant contained twice as much of these two phenolics compared to the aboveground part. Subcritical water extraction of *O. mutabilis* is an environmentally friendly method that can extract selected phenolics at higher concentrations compared to maceration.

## 1. Introduction

The progress of the COVID-19 pandemic since 2019 has highlighted the need for using medicinal plants and/or natural products as promising alternatives to prevent or treat diseases [1]. Plants generally contain many effective bioactive compounds, such as flavonoids and polyphenols [2,3,4,5], essential oils and terpenoids [6,7,8], fatty acids [9,10], and alkaloids [11,12]. In addition, the presence of antioxidants [13,14] as well as antiinflammatory [15,16] and antimicrobial agents [17,18], which have different therapeutic effects, have been reported in many studies. The analysis of bioactive substances in medicinal plants or natural products relies on the use of feasible and effective extraction methods. Optimizing the variables that affect the extraction process (solvent quantity, solvent/plant ratio, temperature, pressure, time, heating rate) is as important as the selection of the extraction method to be used [19]. Response surface methodology (RSM) is an experimental optimization strategy in which mathematical models and statistical analysis are combined to reduce the number of experiments and the use of solvents, and save time. Furthermore, RSM reveals the relationships between the experimental factors and responses [20]. In addition, this method generates functional theoretical equations for response estimates at any desired level within the working range of experimental variables. 

Although there are many extraction methods, the most commonly used are Soxhlet extraction, maceration, and percolation. These methods have important limitations, such as high solvent consumption, long processing times, low extraction efficiencies, and they are not environmentally friendly [21]. Subcritical water extraction (SWE) is an efficient, low-cost, and green alternative method, capable of overcoming these limitations as demonstrated in several cases [22,23,24,25,26]. In SWE, water is used as the extraction solvent at temperatures in the range of 100–374 °C and with enough pressure to maintain the liquid state of water [27]. Although studies using SWE for the extraction of bioactive components from plants have been reported [28,29,30], this is the first time that SWE has been applied for the extraction of bioactive substances from *O. mutabilis*.

The *Boraginaceae* family, which ranks ninth among the families with the highest number of species in Turkey, includes 357 taxa, 34 genera, 325 species, 16 subspecies, and 16 varieties. *Onosma* L., the largest genus of the *Boraginaceae* family, is represented by a total of 150 species worldwide [31,32]. In Turkey, *Onosma* L. is represented by 97 species, four varieties, and one hybrid species, of which 50 species and one type are endemic [33,34]. Many of the *Onosma* species have therapeutic potential, and these plant species are widely used in traditional medicine [35]. Specifically, it is known that *Onosma* species are used in the relief of bladder pain, the treatment of kidney infections, and wound and burn healing [36]. In addition, studies with *Onosma* species have shown that phenolic compounds have anticancer, antiinflammatory, antimicrobial, antioxidant, and wound healing properties [37]. Results related to the extraction of *O. mutabilis* using alternative, environmentally friendly methods are completely missing from the published literature. Furthermore, given the tunable properties of subcritical water, the hypothesis is that it could potentially extract bioactive substances that conventional extraction methods may not extract. 

The rationale of this work was to investigate the extraction of bioactive substances from *O. mutabilis* under subcritical conditions, as an environmentally friendly extraction strategy, and compare its efficiency and identity of extracted substances to conventional extraction methods. Therefore, the specific objectives are the following: (a) to determine the total phenol content and chemical profiles of the extracts obtained by SWE of the aboveground and root parts of *O. mutabilis*, (b) to optimize the SWE process parameters (temperature, extraction time, and plant/water ratio) using RSM and determine the interactions between them, and (c) to compare SWE of the aboveground and root parts of *O. mutabilis* with conventional extraction (water maceration) in terms of the extracts’ composition and total phenolic contents.

## 2. Results and Discussion

### 2.1. Optimum Extraction Yield and Total Phenolic Content

The actual (experimental) and predicted values for the extraction yields and total phenolic content (TPC) can be seen in Table 1. For the aboveground part, the highest extraction yield of 87.19% was obtained at a temperature of 200 °C, 180 min residence time, and a water/plant ratio of 0.25. For the roots, the highest extraction yield of 72.6% was obtained at a temperature at the same set of conditions. Therefore, it appears that the aboveground part contains a significantly higher content of total extractables. 

With respect to the total phenol content, the optimum value of 193.9 mg GAE/g was obtained from the aboveground part at 150 °C, 180 min, and 0.1 water/plant ratio. The roots yielded a lower TPC content of 174.1 mg GAE/g at 200 °C, 105 min, and 0.1 water/plant ratio. However, a comparable TPC (173.1 mg GAE/g) was obtained at the lower temperature of 150 °C; therefore, this can be considered as the experimental optimum temperature for both parts of the plant, having in mind the requirement for energy-efficient extractions at a larger scale. Since the SWE of the aboveground part resulted in a higher yield and higher total phenolic content, it can be considered as the optimum part of *O. mutabilis* in view of large-scale extraction procedures.

### 2.2. Multiple Regression Modelling of the Extraction Yields and Analysis of Variance 

The experimentally obtained data (Table 2) for the aboveground part and roots were subjected to regression analysis and the following empirical relationships between the extraction yield and the independent variables (A: temperature, B: extraction time, and C: water/plant ratio) were obtained

(1) $$Extraction\;yield\;\%\;\left(aboveground\;part\right)=+69.16+11.60A+4.24B-2.30C+5.12AB+7.57AC+5.49BC-2.40A^{2}-2.35B^{2}-2.36C^{2}$$

(2) $$Extraction\;yield\;\%\;\left(roots\right)=+55.00+13.95A+4.53B+2.19C+1.29AB+0.97AC-2.69BC-4.37A^{2}+1.90B^{2}-4.74C^{2}$$

Both Equations (1) and (5) highlight that temperature (A) was the most influential parameter in the extraction process, as shown by the coefficients of 11.60 and 13.95, respectively. Furthermore, the positive sign for these coefficients showed that temperature had a positive linear effect, indicating that higher temperatures resulted in higher extraction yields. This may be attributed to the higher solubility of the essential oil components at elevated temperatures [38]. Furthermore, the vapor pressure of subcritical water should also be considered an important factor for extraction. Higher vapor pressure at higher temperature promotes penetration of the water molecules through the sample matrix, while the higher diffusivity of subcritical water and lower surface tension enhances the transport of the essential oil components into the bulk water phase resulting in higher extraction efficiency [39].

The second most influential parameter was extraction time (B), followed by the water/plant ratio (C). Interestingly, opposite trends were observed for the aboveground part and roots with respect to the water/plant ratio. Increasing the water/plant ratio had a slightly negative impact on the extraction yield of the aboveground part and a slightly positive one for the roots. This may be attributed to the different recalcitrance to degradation of components found in the aboveground part but not present in the roots. This hypothesis will be further investigated later in Section 3.5 where the composition of the extracts is discussed. 

Furthermore, data were analyzed using the variance analysis (ANOVA), and the results are presented in Table 2. The regression coefficient (R^2^) can be used to evaluate the fit of the model. The ANOVA revealed that the aboveground part and root R^2^ values were 0.9796 and 0.9633, respectively. According to these values, the proposed mathematical models account for more than 97.96 and 96.33% of the response’s overall variation for the aboveground part and roots, respectively. The *p*-values were also used to check the significance of each coefficient and display the interaction pattern between the variables. It can be seen from Table 2 that the linear terms for temperature and extraction time were statistically significant (*p* < 0.05) and played an important role in the extraction yields for both parts of *O. mutabilis*. The same applies to the combined parameters (AB, BC, AC); however, the quadratic terms were all statistically insignificant, and can therefore be omitted from Equations (1) and (5). Furthermore, the reproducibility of the model was tested using the coefficient of variation (CV), which is the ratio of the standard error of estimate to the mean value of the observed response (given as percentage). The CV values of 4.15 and 7.57% for the aboveground part and roots, respectively, indicate a highly reproducible model.

**Figure 1:**
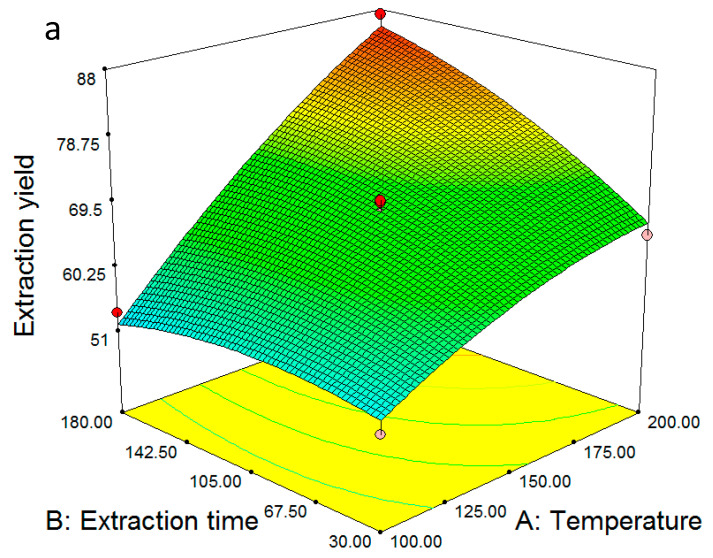

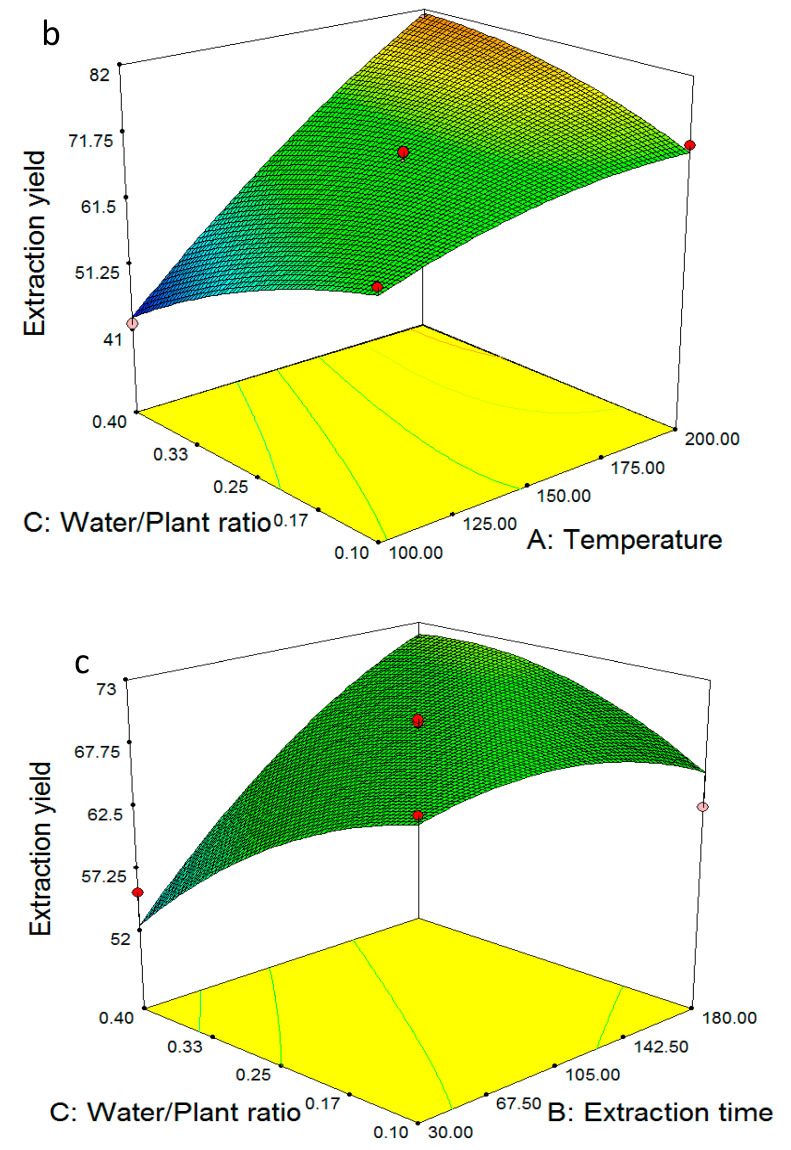

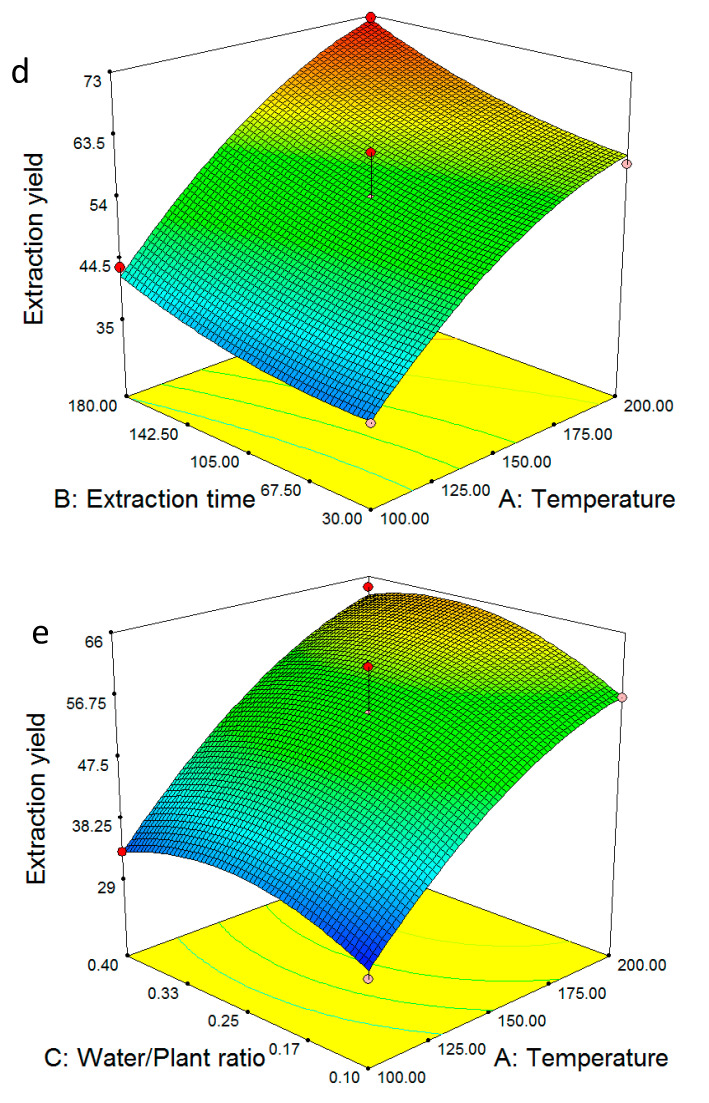

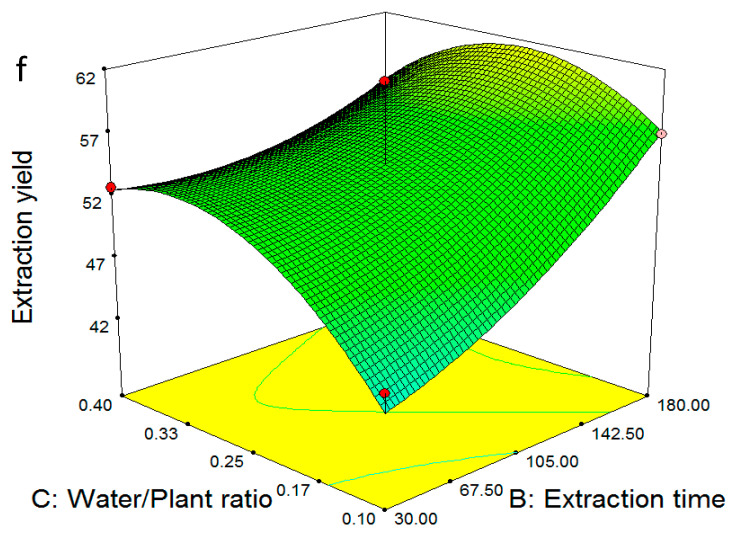
The 3D response surfaces of the extraction yield for the aboveground part (**a**–**c**) and roots (**d**–**f**) of *O. mutabilis*. In (**a**,**d**), the water/plant ratio was kept constant at 0.25. In (**b**,**e**), the extraction time was kept constant at 105 min. In (**c**,**f**), the temperature was kept constant at 150 °C.

The contour plots and the corresponding 3D views provide a valuable insight on the combined influence of the independent variables and their effect on the dependent variable. Figure 1a–c correspond to the extraction yields obtained from the aboveground part. Figure 1a shows that at the lowest temperature of 100 °C, increasing the extraction time had practically no influence on the extraction yield, whereas at the highest temperature of 200 °C, increasing the extraction from 30 to 180 min resulted in ~20% raise in extraction yield. This is an indication that extraction at this temperature is not so much kinetically driven as it is thermodynamically.

In Figure 1b, the rate-determining influence of temperature can be seen again. At the lowest temperature of 100 °C, increasing the water/plant ratio inside the reactor from 0.1 to 0.4 reduced the extraction yield by ~15%. A possible explanation for this is the participation of the water molecules in oxidation and degradation reactions for the extracted components. The opposite trend was observed at the highest temperature of 200 °C, where increasing the water/plant ratio from 0.1 to 0.4 increased the extraction yield by approximately 15%. At these conditions, the water molecules still participated in degradation reactions; however, the rate of extraction surpassed the rate of degradation, resulting in a net raise in the extraction yield. The combined influence of extraction time and water/plant ratio (Figure 1c) was less significant compared to the ones shown earlier. At the lowest water/plant ratio of 0.1, increasing the reaction time from 30 to 180 min had a minimal effect on yield, whereas at the highest water/plant ratio the effect of increasing the reaction time was dramatic, resulting in ~20% higher yield. This observation may be attributed to the fact that more water molecules were able to penetrate the plant matrix and extracted a higher number of components, which masked potential degradation side-reactions. However, a smaller number of water molecules meant a reduced degree of interactions with the solutes and combined with the degradation caused by the longer extraction times, resulting in a lower extraction yield.

Figure 1d–f correspond to the extraction yield obtained from the roots of the plant. In a similar manner to the aboveground part, increasing the temperature from 100 to 200 °C at each and all extraction times, increased the extraction yield by 30% (from ~40 to ~70%, Figure 1d), confirming the critical role of temperature. Regardless of temperature, a longer extraction time had minimal impact on the extraction yield. A similar pattern was observed in the interaction between temperature and water/plant ratio (Figure 1e). At each and all water/plant ratios, increasing the temperature from 100 to 200 °C resulted in more than double the extraction yield (30 to ~62%). This indicates that in the studied range, the number of water molecules did not play a significant role and temperature was the rate-determining parameter. The comparatively minor influence of water quantity in subcritical water processes (extraction and degradation) has been well-established, provided there are enough water molecules to penetrate the solid matrix and carry the solutes of interest to the bulk aqueous phase. Figure 1f shows the interaction between water/plant ratio and extraction time at a constant temperature of 150 °C. At the lower water/plant ratio of 0.1, raising the extraction time from 30 to 180 min led to a ~15% increase in the extraction yield, whereas at a ratio of 0.4 the increase was only ~5%. The reason for this difference is probably the more prominent role of degradation side-reactions at higher water/plant ratios. This hypothesis is further supported by the curvature of the 3D surface at the longest extraction time of 180 min. It can be seen that up to the water/plant ratio of 0.25, the yield increased; however, beyond that value, excess water molecules participated in degradation reactions, thus reducing the yield.

### 2.3. Multiple Regression Modelling of the Total Phenol Content and Analysis of Variance 

The TPC experimental values (Table 1) for the aboveground part and roots were subjected to regression analysis and the following empirical relationships between the TPC and the independent variables (A: temperature, B: extraction time, and C: water/plant ratio) were obtained:

(3) $$TPC\;\left(aboveground\;part\right)=+57.84+18.97A+5.40B-58.95C-3.02AB-14.84AC-18.70BC-15.14A^{2}-0.83B^{2}+44.52C^{2}\;$$

(4) $$TPC\;\left(roots\right)=+72.33+10.16A+1.77B-61.23C-2.90AB-6.34AC-1.20BC-10.41A^{2}+1.07B^{2}+33.18C^{2}$$

Based on the coefficients of each variable, temperature (A) had the most significant positive influence on TPC for both parts of the plant. As in many cases of phenol extraction from environmental matrices, increasing the temperature increases their solubility and thus their content in the extract [40]. Notably, the effect of temperature was more critical in the case of the aboveground part than the roots. The same applied for the extraction time (B), although overall its influence was less significant than temperature. Regarding the water/plant ratio (C), a strong negative dependence was determined, indicating that as the ratio increased, the TPC decreased. This negative dependence may be attributed to either the dilution of phenols as more components from other chemical classes were extracted (such as terpenes, aldehydes, and ketones) or the degradation of phenols in water-participating side-reactions. Taking into consideration the minimal influence of the water/plant ratio on the yield of the total extractables (Table 2, *p*-values of 0.06 and 0.17 for the aboveground part and roots, respectively), and the related published literature, the second hypothesis appears to be more realistic [41].

The ANOVA results are shown In Table 3. The regression coefficients of 98.99 and 99.27% for the aboveground part and roots, respectively, establish the applicability of the proposed model equations. With respect to the significance of each variable, the linear terms for temperature and water/plant ratio were statistically significant (*p* < 0.05) and played an important role in the TPC for both parts of *O. mutabilis*. Contrary to these, the influence of the extraction time was deemed as insignificant for both the aboveground part and roots (*p*-values of 0.16 and 0.52, respectively). The CV values of 12.68 and 8.54% for the aboveground part and roots, respectively, indicate a reproducible model.

**Figure 2:**
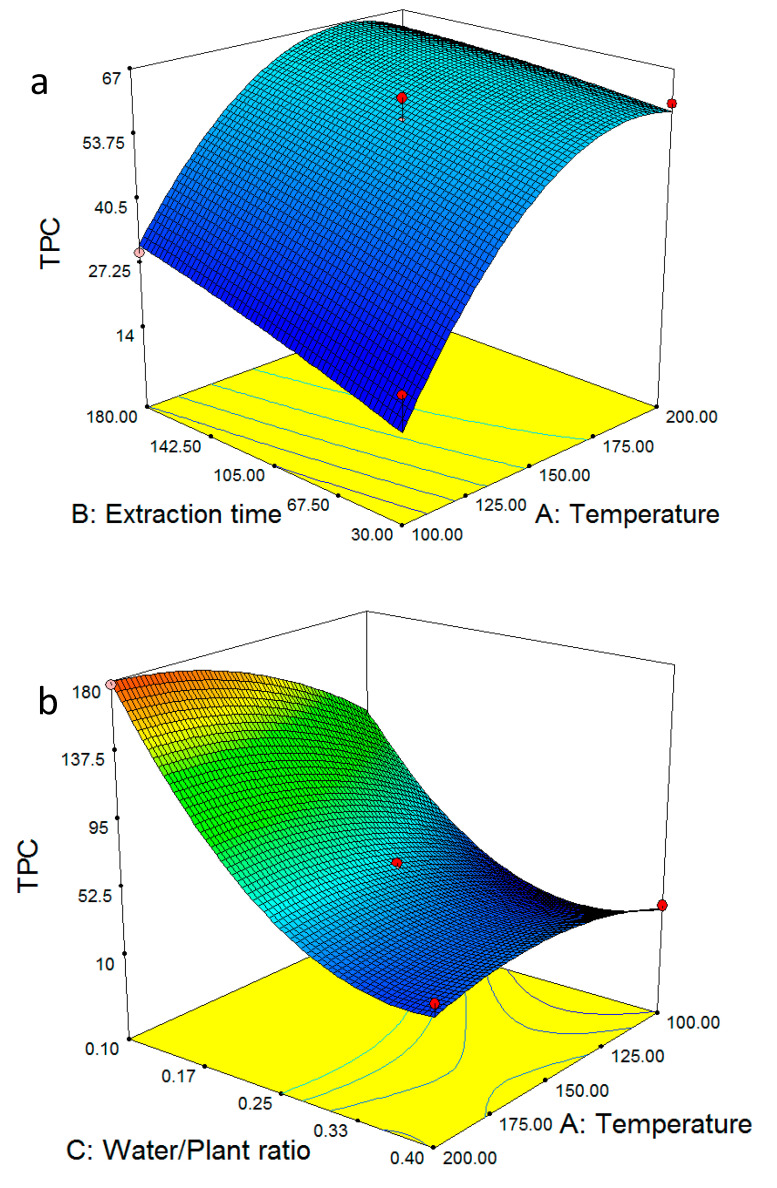

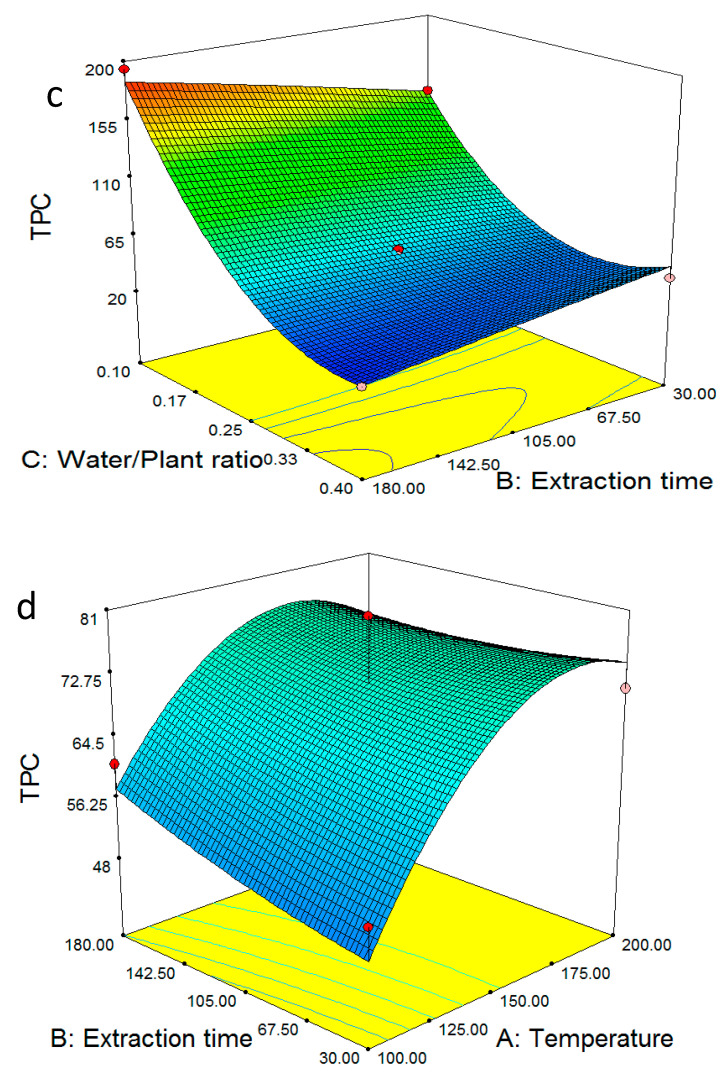

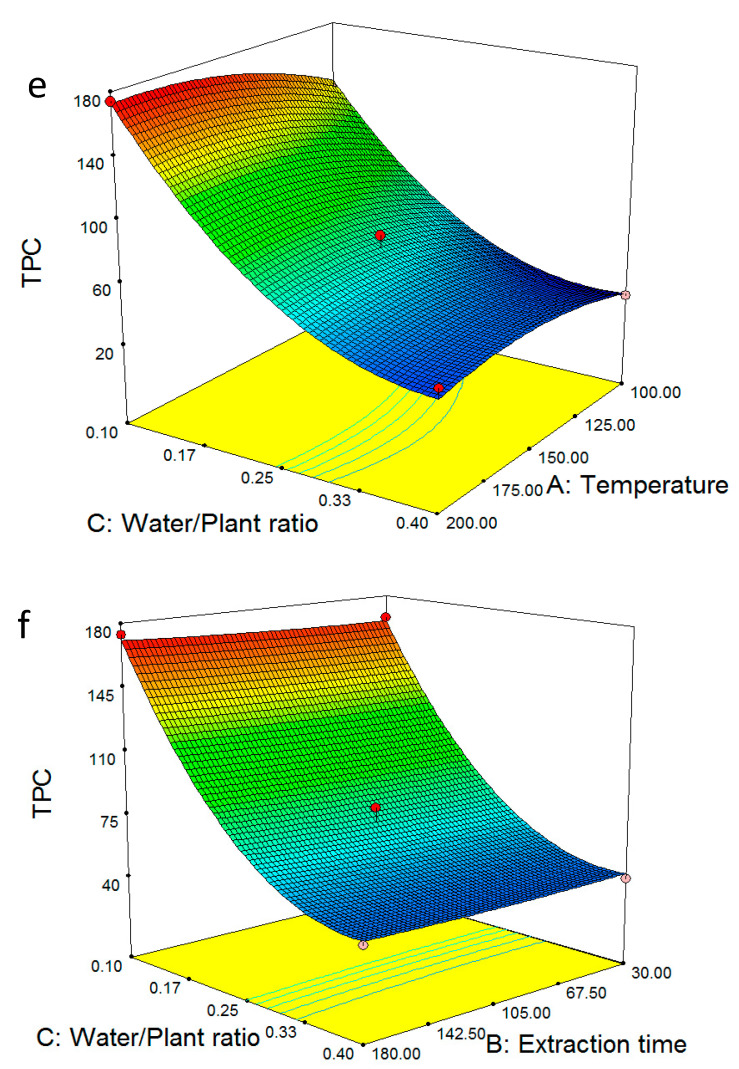
The 3D surface plot results of TPC for the aboveground part (**a**–**c**) and roots (**d**–**f**) of *O. mutabilis*. In (**a**,**d**), the water/plant ratio was kept constant at 0.25. In (**b**,**e**), the extraction time was kept constant at 105 min. In (**c**,**f**), the temperature was kept constant at 150 °C.

The 3D plots of Figure 2 present the interactive effect of the variables on the TPC. Figure 2a shows the effect of extraction time and temperature on the TPC. The significant and insignificant roles of temperature and extraction time, respectively, were further established. At each and all temperatures, increasing the extraction time resulted in an approximately ~5% raise in TPC. On the other hand, at each and all extraction times, doubling the temperature from 100 to 200 °C more than doubled the TPC (from 25–30 to 60–65%). Physical properties such as the dielectric constant of water, which decreases with increasing temperature, influences the SWE of nonpolar phenolics [42]. Nonpolar solutes become more soluble as temperature rises. These findings were in accordance with earlier results on the total phenolic content of subcritical water extracts [43,44]. The flattening of the curvature at 200 °C potentially suggests that beyond this temperature, the TPC is reduced. Compared to the respective graph for the extraction yield for the aboveground part (Figure 1a), the observation is the same at low temperatures but differentiates as the temperature is raised. Given that the extraction yield includes several classes of compounds and not only phenols, it can be hypothesized that the influence of time increases for classes of compounds that are not as readily soluble in SWE as phenols. Therefore, if the extraction target includes other components of interest, the effect of time should not be overlooked. The most striking feature of Figure 2b is the different effect of temperature between the lowest and highest water/plant ratio. Notably, at a ratio of 0.1, raising the temperature increases the TPC, whereas when more water is added in the system (ratio of 0.4), the TPC remains practically unchanged. This is an indication that phenol degradation occurs after extraction with the participation of water molecules, to support the hypothesis proposed in Section 2.1 (Figure 1b).

Figure 2c confirms the minimal role of extraction time within the studied range, whereas it becomes clear that increasing the water/plant ratio has a negative effect on TPC, which comes in good agreement with the degradation hypothesis proposed above. Figure 2d–f, which correspond to the responses obtained by the SWE of the roots, closely follow the patterns observed in Figure 2a–c.

### 2.4. Model Validation

By using the Equations (1)–(3) and (5) and setting the minimum acceptable values for the extraction yield and TPC at 70% and 170 mg GAE/g, respectively, the predicted optimum conditions for each part of the plant and for the extraction yield and TPC were determined and matched the experimentally observed optimum conditions, shown earlier in Table 1. 

Triplicate experiments were conducted at these conditions and the results are shown in Table 4. It can be seen that the experimental values and the predicted values come in very good agreement. Therefore, it is suggested that the models developed can be reliably used to design the SWE experiments of both parts of *O. mutabilis* and maximize the extraction yield and TPC of the extracts.

### 2.5. Chemical Composition of O. mutabilis Extracts

The chemical composition of the extracts obtained from the SWE of *O. mutabilis* were determined using headspace solid-phase microextraction–gas chromatography–mass spectrometry (HS-SPME-GC/MS.) This method has become one of the basic methods for determining volatile compounds’ quality [45]. The most abundant components in the aboveground part of *O. mutabilis* were hydrocarbons, aldehydes, ketones, and alcohols. Our findings are in good agreement with the published literature [46]. A higher number of compounds, especially phenolics, were identified in the extracts obtained from SWE of the aboveground part. These phenolic compounds included guaiacol (6.78%), 2,6-dimethyl-phenol (1.05%), 2-ethyl-phenol (2.05%), 2,3-dimethyl-phenol (0.89%), 4-ethyl-2-methoxy-phenol (2.33%), 3,5-bis(1,1-dimethylethyl)-phenol, (0.71%), (E)-2-methoxy-5-(1-propenyl)-phenol (0.7%), 2-methyl-phenol (3.59%), and 2,4-dimethoxyphenol (1.04%). 3-methyl-butanal (2.11%), 2-Heptanol (2.78%), and 2,4-dimethyl-3-pentanol, (3.38%) were other major compounds. The amount of volatile phenolic components formed in extracts differed between the two different parts of the plant. The guaiacol content was higher (11.78%) in the roots than the aboveground part, highlighting the higher lignin content of the roots. Guaiacol, which has the highest relative abundance among the volatile compounds, exhibits proven pharmacological effects such as high antioxidant and antiinflammatory activity [47,48]. Some pyrazine compounds were also determined such as methyl-pyrazine (1.19%), 2-ethyl-5-methyl-pyrazine (0.7%), and trimethyl-pyrazine (0.8%). Pyrazine and its derivatives, which are widely distributed in nature, have great importance in medicinal chemistry. Studies have highlighted the role of pyrazine nuclei in binding to macromolecular receptors, forming complexes with significant antimycobacterial activity [49].

The most abundant classes of volatile components occurring in the roots were alkanes (35.68%), alcohols (27.54%), and organic acids (13.04%). The extraction of more alkanes and organic acids was observed in the root part compared to the aboveground part. The production of organic acids from the roots is a natural process for most plants and is used for enhancing phosphorus acquisition, aluminum tolerance, and utilizing beneficial rhizobacteria [50]. The concentration of monoterpenes was low in both samples. Pulegone was found in subcritical water extracts, 1.35 and 0.95% in the aboveground part and roots, respectively. Pulegonein, a monoterpene ketone derivative, has been reported to have antibacterial, antioxidant, and antiinflammatory properties [51]. Similar to the aboveground part, SWE of the roots yielded extracts rich in phenolics (28.96%).

For comparison, the volatile phenolic content of the extracts obtained by conventional maceration was investigated. The volatile components of the maceration extracts were different to the subcritical water extracts. Alcohols (54.02%), esters (5.01%), and organic acids (3.29%) were the three main organic classes of compounds in the maceration of *O. mutabilis*’ aboveground part. The maceration extracts of the roots yielded a reduced content of alcohols (22.76%), whereas the content of esters (12.11%) and organic acids (10.90%) was increased compared to the aboveground part. 3,5-bis (1,1-dimethylethyl)phenol, (3.67%) and guaiacol (0.45%) were found only in the maceration extracts of the root.

Principal component analysis (PCA) is a chemometric technique used to analyze the complex chemical composition of the samples using the relative concentrations of the major compound classes determined by GC-MS analysis (XLSTAT software, Lumivero, Denver, CO, USA). The PCA analysis confirmed significant differences in the chemical composition of *O. mutabilis* depending on the extraction method and part of the plant.

**Figure 3:**
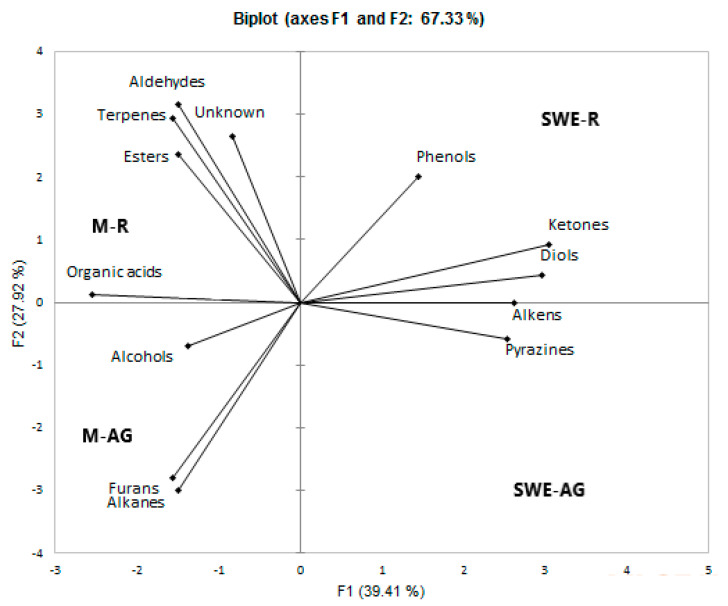
PCA of the main classes of compounds identified. M-AG: maceration aboveground part, SWE-R: subcritical water extraction roots, M-R: maceration roots, SWE-AG subcritical water extraction aboveground part.

In the PCA biplot shown in Figure 3, the extracted classes of compounds were divided into four different areas depending on the extraction method and part of the plant (SWE-AG, SWE-R, M-AG, and M-R). The variance that could be explained was 67.33% (factor 1, 39.42%; factor 2, 27.92%). The compound will have a high abundance value on the positive side of the axis and a low abundance value on the negative side in the PCA. Here, it was found that SWE was closely related to the extraction of phenols, aldehydes, ketones, and pyrazines. The maceration method promoted the extraction of organic acids, alcohols, esters, and terpenes. The alcohols and alkanes were associated with both parts of the plant. As a result, it was established that subcritical water favored the extraction of volatile phenolic compounds compared to maceration.

### 2.6. Method Validation and Quantification of Phenolic Compounds

The linearity, LOD, LOQ, and other method validation parameters are shown in Table 5. Based on the respective equation for each substance, the subcritical water and maceration extracts were quantified and the results are shown in Table 6. The results showed that pyrocatechol and epicatechin were the phenolics with the highest concentration determined after SWE of both parts of the plant. The roots of the plant contained the highest concentration of pyrocatechol (1062 µg/g) and epicatechin (1109 µg/g), whereas the aboveground part contained approximately half these concentrations, 544 and 513 µg/g, respectively. This result contrasts with studies that have reported that *O. mutabilis* species do not contain epicatechin, pyrocatechol, and catechin [52,53]. The concentration of the remaining phenolics obtained from SWE was comparable for both parts of the plant with the exception of quercetin, the concentration of which in the aboveground part was 5-fold that in the roots. Compared to maceration, SWE showed a superior performance for the selected phenolics with the exception of caffeic and ferulic acids where the extracted concentration was slightly reduced. With respect to pyrocatechol and epicatechin, SWE of the roots yielded a much higher concentration compared to conventional maceration, 1062 as compared to 10.2 and 1109 as compared to 23.4 μg/g, respectively. 

## 3. Materials and Methods

### 3.1. Materials 

Folin–Ciocalteu reagent, gallic acid (3,4,5-trihydroxybenzoic acid, abbreviated as GA), anhydrous sodium carbonate (Na_2_CO_3_), were obtained from Sigma-Aldrich (St. Louis, MO, USA). Reagents pyrocatechol, catechin, caffeic acid, epicatechin, *p*-coumaric acid, ferulic acid, and quercetin were of analytical grade and supplied from Merck (Darmstadt, Germany). The plant species *O. mutabilis* was collected in Mersin, Turkey, and identified by Dr. Riza Binzet (Location: C5 Mersin, Mersin-Gözne, around Darısekisi, rocky slopes and scrub area, 36°58′10.91″ N 34°34′11.79″ E, altitude of 780 m). Ultrapure water (18 MΩ cm at 25 °C) was provided by a Millipore Milli-Q Advantage A10. 

### 3.2. Sample Preparation and Extraction Procedures

The collected plants were air-dried in the shade at room temperature (25 °C) for three weeks. Then the aboveground part (stem and leaves) and the roots were separated and reduced to powder separately with a blender (8011ES Model HGB2WTS3 400 W), before storing them in glass bottles at room temperature. Two extraction methods were applied: SWE and hot water maceration. SWE was carried out at laboratory scale in a Teflon-coated, homemade stainless steel reactor (150 mL capacity) fitted with a magnetic stirrer. The pressure inside the reactor was built with N_2_ gas and fixed at 30 bar to keep the water in liquid state. The stirring speed was set at 400 rpm. The extracts obtained after extraction were vacuum-filtered through Whatman No.1 filter paper and the filtrates were stored in glass vials at 4 °C for further analysis.

The method of maceration with hot water has been established as a conventional extraction method for plant tissues (extraction time 180 min, extraction temperature ~100 °C) [54]. The parameters of the SWE method were optimized as described in Section 3.3. The extraction yield for both SWE and maceration was calculated as follows:

(5) $$extraction\;yield\;\left(\%\right)=\frac{dry\;plant\;weight\;\left(g\right)\;before\;extraction-dry\;plant\;weight\;\left(g\right)\;after\;extraction}{dry\;plant\;weight\;\left(g\right)\;before\;extraction}\times 100$$

### 3.3. Optimization of SWE Process

Optimization of the SWE conditions were conducted according to the Box–Behnken design (BBD). RSM was then used for the statistical processing of the experimental data (Design-Expert software, version 7, StatEase, Minneapolis, MN, USA). The effects of the independent variables: temperature (100–200 °C), reaction time (30–180 min), and water/plant ratio (0.1–0.4), were investigated. The experimental design is shown in Table 7. It has been established that during SWE, pressure has a minor influence compared to temperature; therefore, it was not one of the studied variables [55,56].

### 3.4. Determination of Total Phenolic Content

The total phenolic contents of the aboveground and root extracts obtained by subcritical water extraction and maceration were determined by the standard Folin–Ciocalteu method [57]. The calibration curve was prepared in the concentration range of standard gallic acid from 25 to 800 mg/L. Freshly prepared 1 mL of Folin–Ciocalteu reagent and 1 mL of diluted (1:10) sample solution were mixed and stored in the dark for 5 min. Then, 2 mL of sodium carbonate (20%, *w*/*v*) were added. This mixture was vortexed and adjusted to 6 mL by adding 2 mL of ultrapure water. After 30 min, absorbance was measured at 714 nm using a UV-visible spectrophotometer (UV-1601, Shimadzu, Japan). The concentration of total phenolic content was expressed as milligram gallic acid equivalents. The results were given as the average of three measurements.

### 3.5. Analytical Methods

Fourier transform infrared (FTIR) spectroscopy and solid-phase microextraction coupled with gas chromatograph/mass spectrometry (SPME-GC-MS) were used to monitor the phenol identity and composition. The FTIR spectra of samples were obtained between 4000 and 450 cm^−1^ using a JASCO FTIR-ATR spectrophotometer. The volatile phenolic compounds in the extracts were analyzed using a 7890 Agilent gas chromatograph and a 7010B MS detector equipped with a DB-WAX (60 m length 0.25 mm i.d. 0.5 m thickness) capillary column. Briefly, 3 mL of extracts were put into a 20 mL headspace vial and equilibrated at 60 °C for 15 min. The volatiles were then extracted for 30 min by a 1 cm solid-phase microextraction fiber assembly (CAR/PDMS/DVB-fiber (Supelco), 50/30 µm) with continuous stirring at 60 °C (250 rpm). Thermal desorption was carried out for 5 min at 250 °C. The column temperature was initially held at 40 °C for 4 min, then raised to 90 °C at a rate of 3 °C/min, then to 130 °C at a rate of 4 °C/min and held for 4 min, then raised to 240 °C at a rate of 5 °C/min and held there for 8 min. The NIST 14 library was used to identify the volatile substances in the extracts. 

The quantification of selected phenolic compounds (gallic acid, pyrocatechol, catechin, caffeic acid, epicatechin, *p*-coumaric acid, ferulic acid, and quercetin) of the extracts were determined by using high performance liquid chromatograph coupled with diode array detector at a wavelength of 278 nm (HPLC-DAD, Shimadzu Nexera 2). The column was Inertsil ODS-4 C_18_ (250 mm × 4.6 mm, 5 μm). The mobile phase consisted of 0.2% acetic acid in water (A) and methanol (B) using a gradient elution as follows: 0–0.1 min, 5% B; 0.1–3 min, 95% B; 3–18 min, 20% B; 18–20 min, 20% B; 20–30 min, 40% B; 30–40 min, 50% B; 40–50 min, 100% B; and 50–55 min, 5% B for equilibration of the column. The column temperature was maintained at 30 °C. In total, 20 µL of extract were injected into the column in each run. The flow rate was 1 mL/min [58]. Analytes in each subcritical water extract were identified by comparing their retention times and UV–vis spectra with those of standard compounds. Individual stock solutions of standard phenolic compounds were prepared in methanol (2000 mg/L), and their mixtures to plot the calibration curves ranging from 0.5 to 100 mg/L were made in methanol–water (50:50, *v*/*v*). The phenolics were quantified using an external standard calibration. Limit of detection (LOD), limit of quantification (LOQ, mg/L), and the coefficient of correlation (R^2^) are shown in Table 5. Results were means of triplicate injections and expressed as μg/100 g dry sample.

## 4. Conclusions

Based on successful stories reported in the past, the interest for extracting bioactive substances from plants and other naturally occurring matrices has remained high. The cosmetic and pharmaceutical industries have developed processes that aim at extracting such substances at as high a yield and purity as possible. However, conventional extraction methods have raised environmental concerns and ‘green’ alternative procedures of equal performance are required. In this framework, subcritical water extraction has shown its potential in a wide range of cases, achieving high yields and selective extractions, and at the same time, avoiding costly and hazardous organic solvents. In this work, it was demonstrated that SWE can be comparably more efficient than maceration for the extraction of bioactive compounds from the aboveground part and roots of the plant. The added advantage of SWE is that it can be fine-tuned to extract specific substances, by changing the working temperature, water/plant ratio, and extraction time, thus avoiding the formation of heavily crude extracts that require extensive, post-extraction cleaning steps. Based on the optimization performed in this study, future work will focus on the scaling up of the process and verification of the laboratory results.

## Figures and Tables

**Table 1:** The actual and predicted responses for the extraction yield and total phenol content, based on the 3-factor and 3-level Box–Behnken design.

Experimental Design Parameters			Aboveground Part				Roots			
Temperature (°C)	Extraction Time (min)	Water/Plant Ratio	Extraction Yield (%) Predicted	Extraction Yield (%) Actual	TPC mg GAE/g	TPC mg GAE/g Actual	Extraction Yield (%) Predicted	Extraction Yield (%) Actual	TPC mg GAE/g Predicted	TPC mg GAE/g Actual
200.00	105.00	0.4	81.28	80.13	32.42	40.06	63.00	64.21	37.68	44.01
100.00	105.00	0.4	42.94	41.88	24.15	26.18	33.16	33.25	30.05	28.74
150.00	180.00	0.4	71.88	71.21	29.28	28.94	56.2	54.69	45.91	43.84
100.00	105.00	0.1	62.66	63.81	112.37	104.73	30.72	29.51	139.83	133.50
100.00	180.00	0.25	51.93	53.65	31.33	29.64	41.82	43.24	57.50	60.87
200.00	30.00	0.25	66.67	64.94	58.47	60.16	60.65	59.23	74.28	70.91
150.00	30.00	0.4	52.43	55.30	55.90	46.57	52.51	52.72	44.77	41.82
100.00	30.00	0.25	53.69	51.87	14.49	21.78	35.34	35.04	48.16	52.42
200.00	105.00	0.1	70.74	71.79	179.99	177.96	56.67	56.58	172.83	174.14
150.00	105.00	0.25	69.16	69.61	57.84	56.07	55.00	50.00	72.33	80.24
150.00	30.00	0.1	68.00	68.67	136.38	136.72	42.75	44.26	164.84	166.91
150.00	105.00	0.25	69.16	69.86	57.84	61.27	55.00	61.08	72.33	67.49
150.00	105.00	0.25	69.16	68.02	57.84	56.18	55.00	53.91	72.33	69.27
200.00	180.00	0.25	85.37	87.19	63.22	55.93	72.3	72.6	72.01	67.75
150.00	180.00	0.1	65.49	62.61	184.58	193.91	57.19	56.98	170.77	173.13

**Table 2:** Variance analysis for the extraction yields of the aboveground part and roots of *O. mutabilis*.

Extraction Yield (Aboveground Part)	Sum of Squares	df	Mean Square	*F*-Value	*p*-Value
Model	1771.50	9	196.83	26.69	0.0011
A—temperature	1077.41	1	1077.41	146.12	<0.0001
B—extraction time	143.48	1	143.48	19.46	0.0069
C—water/plant ratio	42.14	1	42.14	5.71	0.0624
AB	104.76	1	104.76	14.21	0.0130
AC	229.07	1	229.07	31.07	0.0026
BC	120.67	1	120.67	16.37	0.0099
A^2^	21.23	1	21.23	2.88	0.1505
B^2^	20.44	1	20.44	2.77	0.1568
C^2^	20.62	1	20.62	2.80	0.1554
Residual	36.87	5	7.37		
Cor Total	1808.37	14			
R^2^	0.9796				
CV %	4.15				
Extraction yield (roots)					
Model	1965.28	9	218.36	14.57	0.0044
A—temperature	1556.26	1	1556.26	103.81	0.0002
B—extraction time	164.35	1	164.35	10.96	0.0212
C—water/plant ratio	38.46	1	38.46	2.57	0.1701
AB	6.68	1	6.68	0.45	0.5339
AC	3.78	1	3.78	0.25	0.6368
BC	28.89	1	28.89	1.93	0.2238
A^2^	70.58	1	70.58	4.71	0.0822
B^2^	13.37	1	13.37	0.89	0.3883
C^2^	82.86	1	82.86	5.53	0.0655
Residual	74.96	5	14.99		
Cor Total	2040.24	14			
R^2^	0.9633				
CV %	7.57				

**Table 3:** Variance analysis for the total phenol contents of the aboveground part and roots of *O. mutabilis*.

TPC (Aboveground)	Sum of Squares	df	Mean Square	*F*-Value	*p*-Value
Model	41,856.44	9	4650.72	54.20	0.0002
A—temperature	2879.53	1	2879.53	33.56	0.0022
B—extraction time	233.04	1	233.04	2.72	0.1603
C—water/plant ratio	27,796.69	1	27,796.69	323.94	<0.0001
AB	36.55	1	36.55	0.43	0.5428
AC	880.66	1	880.66	10.26	0.0239
BC	1399.17	1	1399.17	16.31	0.0099
A^2^	845.86	1	845.86	9.86	0.0257
B^2^	2.54	1	2.54	0.030	0.8701
C^2^	7319.23	1	7319.23	85.30	0.0002
Residual	429.04	5	85.81		
Cor Total	42,285.47	14			
R^2^	0.9899				
CV %	12.68				
TPC (Root)					
Model	35,735.85	9	3970.65	75.22	< 0.0001
A—temperature	825.62	1	825.62	15.64	0.0108
B—extraction time	24.96	1	24.96	0.47	0.5222
C—water/plant ratio	29,995.72	1	29,995.72	568.26	<0.0001
AB	33.75	1	33.75	0.64	0.4602
AC	160.83	1	160.83	3.05	0.1413
BC	5.75	1	5.75	0.11	0.7548
A^2^	400.28	1	400.28	7.58	0.0401
B^2^	4.19	1	4.19	0.079	0.7894
C^2^	4063.80	1	4063.80	76.99	0.0003
Residual	263.93	5	52.79		
Cor Total	35,999.78	14			
R^2^	0.9927				
CV %	8.54				

**Table 4:** Validation of the proposed models describing the extraction yield and TPC for the aboveground part and roots, based on triplicate extractions.

	Aboveground Part		Roots	
	Predicted	Experimental	Predicted	Experimental
Extraction yield (%)	72.78	71.61 ± 0.66	67.08	64.48 ± 0.78
TPC mg GAE/g	193.9	193.63 ± 0.40	173.96	173.13 ± 0.91

**Table 5:** Analytical parameters and calibration data for phenolic compounds.

Compound	R^2^	Equation	Linearity (mg/L)	Reproducibility RSD (%)	Repeatability RSD (%)	Sensitivity	
						LOD (mg/L)	LOQ (mg/L)
Gallic acid	0.9994	Y = 69029x − 36592	0.5–50	0.120	4.892	0.010	0.032
Pyrocatechol	0.9998	Y = 32014x − 9886.1	0.5–100	0.765	2.051	0.014	0.048
Catechin	0.9959	Y = 11231x − 7354.7	0.5–100	0.907	2.201	0.076	0.253
Caffeic acid	0.9977	Y = 52828x − 66606	0.5–100	0.282	1.260	0.005	0.017
Epicatechin	0.9993	Y = 14059x − 6188.7	0.5–50	1.821	1.839	0.029	0.098
*p*-coumaric acid	0.9998	Y = 89753x − 30217	0.5–100	0.072	1.136	0.008	0.025
Ferulic acid	0.9998	Y = 63350x − 22113	0.5–100	0.260	1.443	0.002	0.006
Quercetin	0.9999	Y = 41175x − 19407	0.5–100	3.394	7.219	0.051	0.171

**Table 6:** Quantification of *O. mutabilis* extracts.

	µg/g Extract			
	SWE Aboveground	SWE Roots	Maceration Aboveground	Maceration Roots
Gallic acid	39.969 ± 4.40	37.02 ± 0.33	12.18 ± 2.87	9.22 ± 0.24
Pyrocatechol	544.42 ± 68.41	1062.78 ± 114.29	28.93 ± 10.37	10.22 ± 2.65
Catechin	163.99 ± 0.42	145.08 ± 23.10	128.25 ± 2.80	31.54 ± 1.12
Caffeic acid	33.92 ± 10.38	33.58 ± 12.21	64.05 ± 5.382	58.11 ± 4.93
Epicatechin	513.69 ± 55.57	1109.94 ± 37.08	72.72 ± 5.93	23.41 ± 6.01
*p*-coumaric acid	65.57 ± 13.61	59.40 ± 15.14	63.09 ± 0.56	27.04 ± 2.71
Ferulic acid	14.02 ± 5.20	6.68 ± 1.59	5.57 ± 1.76	95.69 ± 1.43
Quercetin	67.55 ± 20.95	15.87 ± 2.38	19.52 ± 3.08	6.91 ± 0.21

**Table 7:** Optimization of the subcritical water extraction of *O. mutabilis*.

Variables	Symbols	Codes		
		−1	0	1
Temperature/°C	A	100	150	200
Extraction time/min	B	30	105	180
Water/plant ratio	C	0.1	0.25	0.4

## Data Availability

Data are contained within the article.
